# From Poland with love: Politics of queerness in the time of neoliberal post-socialism (The case of Michał Witkowski)

**DOI:** 10.12688/openreseurope.19923.1

**Published:** 2025-07-08

**Authors:** Błażej Warkocki

**Affiliations:** 1 Adam Mickiewicz University in Poznan Faculty of Polish and Classical Philology, Poznań, Greater Poland Voivodeship, Poland

**Keywords:** queer, LGBT, political novel, Central and Eastern Europe, Witkowski, Lovetown, neoliberalism

## Abstract

The article presents Michał Witkowski’s novel *Lubiewo* ( *Lovetown*, 2005) as an unconsciously political novel. In order to do so, I take a critical approach to the basic epistemological distinction contained in this novel between *gej/ciota* (gay/faggot) by analysing subsequent novels that refer to *Lovetown*, in particular *Fyn fund cwancy*ś ( *Twentyfive*, 2015) and a privately published autofictional novel entitled *One Hundred Dogs for Killing a Crab. A Cuban Diary* (circa 2018). The article argues that the translation of this distinction (gay/ciota) into the ‘queer versus gay’ opposition, recognisable in theory (especially in the US), leads to misinterpretation and distorts the Eastern European context of economic transformation. Deconstructive analysis shows that the political aspect of Witkowski’s work can be found particularly in his critique of Polish hegemonic masculinity and his critique of the economic dimension of Poland’s neoliberal transformation.

In this essay, I analyse Michał Witkowski’s *Lubiewo* (2005 ^1^; Eng. *Lovetown*, 2010 ^2^), one of the most important queer novels from Poland (and perhaps from Central and Eastern Europe as a whole) in the post-1989 period, which Michał Buchowski has called “neoliberal post-socialism” ^3^. I will also look at his sequels, which are set in German-speaking countries (mainly Austria and German-speaking Switzerland) and Cuba. In my opinion, they suggest that the author acts as if he would not or could not detach himself from his debut novel; or even, to put it another way, as if affect could not detach itself from its subject. Thus, I will present *Lovetown* as a ‘primal scene’ insofar as it records the fragmentation of a trauma that will recur in Witkowski’s later works, particularly in *Fynf and cwancys* (2015; *Twenty-Five*; the title is a transliteration of the Polish pronunciation of the German number 25), which is literally a development of one of the parts from *Lovetown*. An important, if not obvious, continuation will also be a text published in private, non-institutional circulation – *Sto psów za zabicie kraba. Dziennik kubanski* ( *One hundred dogs for killing a crab. Cuban diary*, ca. 2018). The book is out of print, has not been officially published and can only be purchased as a PDF directly from the author. All these texts have the same autofictional protagonist Michaśka (Michał) and his specific attitude towards reality. The framework of the analysis is formed by the East/West distinction as ‘projection surfaces’, the semi-peripherality of Eastern Europe and the transformation of Poland in the 1990s.

*Lovetown* is not a political novel in the conventional sense. Rather, it became political because of the socio-political context of its time, in particular due to the wave of LGBT emancipation in Poland in the first decade of the twenty-first century. The author renounced and still renounces any political involvement. And yet his novel – with reference to the theory of Jacques Rancière ^4^ – shifts the visible to negative experiences. At the same time, the novel rejects the emancipatory narrative of its time, which has its own, not always explicitly articulated reasons. This article is an attempt to understand these reasons and thus to uncover the political unconsciousness of the novel.

## Screens of Fantasy

Immediately after its publication in Poland, *Lovetown* attracted a great deal of attention, and not only among literary critics. This kind of story about non-heteronormative people had never been told in Poland before. All the more so as homosexual themes were still strictly taboo in Polish cultural production until the end of the 1990s. It was not until the first decade of the twenty-first century that things changed radically. This was symbolised by the 2003 exhibition “Let Them See Us” – a collection of around a dozen photos of same-sex couples by artist Karolina Breguła, which were shown in art galleries, but also (in two cases) on billboards in major Polish cities. The couples in the photos were usually young, smiling and metropolitan, but the billboards were nevertheless vandalised by members of the nationalist movement. At this time there were also pride marches and equality parades, which were sometimes banned. During the 2005 Equality March in Poznań, for example, the police arrested 65 people. Nevertheless, activism and repression have increased the visibility of LGBT people in the country. Finally, the rights of LGBT people were discussed in the mainstream press. In a word, homosexuality broke into the public space and public discourse in Poland in the 2000s.

This forms the larger context in which *Lovetown* was published in 2005. However, Michal Witkowski’s aim was not to convey a positive image of queerness but to use a strategy of shock and disgust. Literary critics generally welcomed the book while neutralising it. From this point of view, the novel was not about the people around us, but about ‘distant and exotic Others’. For the critics, Witkowski’s characters (‘cioty’) were supposed to represent a world that was disappearing, namely the People’s Republic of Poland, although the novel obviously also referred to times of economic transition.

The heterogeneous novel *Lovetown* depicts the 1980s ^5^ and 1990s without 1989 representing a major break. The characters notice the social change when shopping centres emerge in place of the traditional cruising sites, much like the characters in Joanna Bator’s novel *Sand Mountain* (2009), who notice the system change when their workplaces are closed. Furthermore, *Lovetown* sets in motion two complementary phantasms of time, located on the East-West line, which are to be treated as specific screens of projection in the psychoanalytical sense, i. e. the subject projects unacceptable impulses onto the cultural text. The first, which is treated by the author as a negative point of reference, centres on the American television series *Dynasty*, while the second, which is mentioned less frequently but – this is an apparent paradox – much more positively, concerns a popular German book whose impact began in the 1980s and continues almost uninterruptedly to the present day, namely *Wir Kinder vom Bahnhof Zoo* (1978 ^6^; *Zoo Station: The Story of Christian F*.) by Christiane F. (Felscherinow), the first Polish edition of which was published in 1987. However, this connection is not a mere intertextuality, but the activation of fantasies recognisable to people of Witkowski’s generation. Perhaps these fantasies have a much more universal meaning and characterise the phantasmatic dimension of Poland’s socio-economic transformation. These fantasies should be understood here according to the guidelines of the Polish literary historian Maria Janion, i. e. according to the Romantic formula that reads: “The Dreamer: (s)he is where (s)he is not; and (s)he is not where (s)he is” ( 7 on page 30). This paradoxical sentence describes the rather obvious status of the dreamer, who sees fantasies as much more real than the reality (s)he inhabits.

The American series *Dynasty*, which has been broadcast on Polish television since the early 1990s – and thus in the last period before the technological revolution – with a delay of a decade compared to the USA, is a specific screen of projection whose meaning in the transformative 1990s is quite easy to decipher. In all likelihood, viewers identified with the ideologies and characters presented on the screen. In the days of early Polish capitalism, which was decidedly neoliberal, viewers imagined they were the Carringtons, American oil moguls living on a Byzantine estate managed by a majordomo. And the wealth that should have become their phantasmatic selves thanks to the transformation had a Protestant moral dimension (quite unlike *Succession* ^8^, the more recent counterpart to *Dynasty*, in which the wealth of American media moguls running a democracy is no longer portrayed in the same ostentatious way and the view of capitalism is already clearly cynical). In other words, in the 1990s, the American high-budget soap opera offered an obvious picture of capitalist *jouissance* in Poland, which we could all hope to enjoy to the fullest.

The second fantasy is decidedly darker and is invoked less frequently. In Witkowski’s recently published *Autobiography* (2023), however, it clearly appears in the form of a ‘forbidden’ book and a signpost that can almost be understood geographically. *Zoo Station*, a youthful memoir that Witkowski mentions in *Autobiography*, describes the hardships of growing up, love, a fascination with David Bowie and addiction, prostitution and death, often recounting the deaths of teenagers. This story has now become a constantly retold myth itself (and that’s exactly how it is treated in its latest remake, a 2021 German Amazon series). All this takes place in the most westernised city in Central and Eastern Europe, Berlin, which forms the basis for a peculiar artistic and cultural phenomenon that Agata Pyzik calls “berlinism” ( 9 on page 89) in her political essay *Arm aber sexy. Culture Clashes in Europe East and West*. This essayistic term describes the fascination of many artists with West Berlin: its aesthetics, its brutalism and, of course, the Wall.

In the narrative of berlinism, creativity is combined with nostalgia and, not infrequently, self-destruction. A shadow of this fantasy can also be found in *Lovetown*: “I, who back then confused art with smoking, who confused being an artist with drinking, who confused writing with being a whore, with autumn, for everything. It was 1988. [...] I must have been fifteen” ( 2 on page 37). Of course, both *Lovetown* and *Twenty-Five* are not literally set in Berlin, which only provides the direction of the fantasy (‘West’). Moreover, *Zoo Station* can be understood as the opposite, dark side of *Dynasty*: a story about outcasts from society who are simultaneously losers in a regime of capitalist productivity.

*Lovetown* should be interpreted in the context of these two screens of fantasy. Since its publication, Witkowski’s novel has become one of the most important literary texts written in Poland after 1989 that deal with (male) homosexuality. Furthermore, the protagonists of the novel – in their dominant reception as obsessed ‘distant Others’ – were supposed to be particularly representative of the Polish People’s Republic or ‘communism’. Therein lies a certain paradox, for *Lovetown* largely refers to the transitional period, the transformative 1990s, and in any case does not, as mentioned above, sketch the sharp turn of 1989 that we like to imagine in the historical order. And yet, due to its generally nostalgic or perhaps ‘ostalgic’ character, it became the queerest novel not only from Poland, but perhaps even from Eastern Europe as a whole. The reference to ‘communism’, which was juxtaposed with the new early capitalist times, only deepened this perception, as ‘communism’ and ‘Easternness’ functioned as synonyms in this discourse.

## Local distinction and its global translation

One of the most important distinctions used in the story was the contrast *gej/ciota* (gay/faggot). It was reinforced paratextually by the statements of the author himself, who set out the distinction in a virtual afterword to *Lovetown* entitled *Pedalstwo a dominujacy dyskurs medialny* ( *Being a Fag and the Dominant Media Discourse* ^10^), which first appeared online and was later reprinted in the book *Polish Literature 1989–2009* edited by Piotr Marecki. The term ‘fag’ was meant to be associated with something primal, visceral, communist, ours. ‘Gay’, on the other hand, was foreign, Western, actually German. As Witkowski himself confirmed in an interview: “I hate the word gay, which I associate with Steven from *Dynasty*. It’s a product of politically correct mass culture [...]” ( 11 on page 27; emphasis in original).

This opposition *gej/ciota* can easily be translated into the American political and theoretical opposition ‘gay versus queer’. It was precisely in this sense that it appeared in one of the most interesting interpretations of *Lovetown*. In her book *Normy widzialności. Tożsamość w czasach transformacji* ( *Norms of Visibility. Identity in Times of Transformation*), Magda Szczęśniak reconstructs the counter-public homosexual discourse since 1989, i. e. mainly (selected) periodicals, and understands *Lovetown* as a summarising or almost over-conscious allegory of non-heteronormativity in Poland at the time of political transition. As Szczęśniak writes: “Witkowski’s prose will become, by the end of this section of the book, an example of a tactic of resistance to normalization politics, a tactic involving a radical turn to local practices of non-normative sexuality” ( 12 on page 174). Witkowski’s heroes thus win the battle of radicalism against the ‘gays’, here epitomised by Steven from *Dynasty*. The primary matrix of distinction, however, consists of American identity distinctions and American identity politics that function *per analogiam*, namely: queer versus LGBT.

As Szczęśniak’s interpretation is one of the more intriguing attempts to read *Lovetown*, it is all the more interesting to consider its limitations, as they are fairly symptomatic of the wider reception of the book. It is worth noting that the author engages in a kind of ‘romanticisation’ of the subject of her analysis, which leads to significant blind spots. For example, the protagonists of *Lovetown* look for men in parks and railway station toilets who are “more or less willing to have sex” ( 12 on page 259). Anyone who has read Witkowski’s novel will remember descriptions not only of obvious sexual violence, but also of rape and possible murder. So the formula “more or less” seems like a bizarre euphemism, impossible in a heterosexual context where it would be unlikely that anyone would describe sexual violence in this way. For some of the characters Witkowski introduces in *Lovetown* are – it is difficult to put it any other way here – simply criminals, and the romanticisation of “local practices of non-normative sexuality” ( 12 on page 174) serves no useful purpose and can sometimes even be counterproductive. For ultimately, the author’s main interpretative distinction, “gays versus queers” (to recall the formula from the title of her book’s chapter), renders invisible the framework that constructs this binary, namely hegemonic Polish masculinity. This is what the struggle is about, this is the phantasmatic lack that this binary tries to hide. In other words, both Witkowski and especially his interpreter focus primarily on the internal opposition (gay/ciota, translated as ‘gay versus queer’) and fail to recognise that this is the result of a broader, complex phenomenon, namely Polish hegemonic masculinity. They also ignore the fact that a coherent critique of normativity should start right here.

This blind spot is also characteristic of the academic gender discourse at the time when a comprehensive conceptual toolbox was being developed in transgender studies. It is all the more surprising that the author does not use it at all and retains potentially offensive words ( *pedały*) without making the slightest effort to use neutral terms (such as trans- or cismasculinity, non-binary). It seems that the reason for this lies above all in the desire to preserve and justify the “gays vs. queers” division introduced or even initiated in *Lovetown* and to perpetuate its potential epistemological consequences. The use of categories associated with the conceptual repertoire of transgender studies would shift the analysis into the realm of trans and cisgender and ultimately address hegemonic Polish masculinity, which is the broadest analytical framework of *Lovetown*.

In this respect, we can once again observe the change that has taken place in Poland over the last two decades since the novel was published. Witkowski’s characters transcend heteronormativity not only on a sexual but also on a gender level. They are rarely completely cisgender; yet despite this fact, they are almost never analysed through the category of transgender (or non-binary). This language only emerged more broadly after the novel was published, but as early as the 2010s (although it existed earlier in the humanities, of course: *A Transgender Studies Reader* was published in 2006 ^13^). This process ran almost parallel to the retreat from a kind of ‘negativity’. The queer politics of the first decade of the twenty-first century in Poland often relied on Judith Butler’s concept of subversion and reinterpretation of pejorative words, which is much less popular and rather rarely used today ^14^. The word *ciota*, which was common in literary discourse at the time, would probably refer to a non-binary or non-cis person today.

## After *Lovetown*

In 2015, Michal Witkowski published another novel, a kind of spin-off of *Lovetown*, which also builds on the autobiographical mythology he had already created. It is called *Twenty-Five*. Here Witkowski developed one of the *Lovetown* episodes entitled *Dianka*, the story of sixteen-year-old Milan from Bratislava, who flees from home to Vienna – to the West – driven by fascination and the desire for a different life. The “West” serves as the screen for the young protagonist’s projections. Life “over there” is supposed to be more beautiful, more colourful, more emancipatory and, of course, – much richer. Unfortunately, the young Eastern European is quickly disappointed. Dianka very quickly “had a taste of the unique and inimitable flavour that the West acquires when you don’t have a penny to your name” ( 2 on page 140), but also because the only job for the young Milan in Vienna was prostitution at the Karlsplatz-Oper station around which “tons of teenage Poles, Czechs, Romanians and Russians, [...] and geriatric Austrians, too, of course” ( 2 on page 132) bustled. In his interviews and television programmes, Michal Witkowski hinted at an autobiographical underpinning to this plot by unmistakably suggesting that Milan from Bratislava could be Michał from Wrocław. And it is precisely this motif that was developed in *Twenty-Five* as an entire novel and not just as a short episode. The novel describes the milieu of young adults from Eastern Europe who prostitute themselves in Vienna and Zurich in Witkowski’s characteristically bittersweet style. The book was quite well received and was recognised above all as a critique of modern capitalism ( 15 on page 2015).

What was overlooked, however, was the actual construction of the novel, which is based on two protagonists who are connected in a certain way. Not one, as one might have expected. Not three, five or eight either. Two. One is a first-person narrator, who is also the main character and focalized in the third person – Dianka, to whom the former often gives advice. The division of functions between them is clear. After a few missteps at the beginning of the novel, the first-person narrator becomes a winner in the brutal world of young Eastern European sex workers: He gains a steady, chic client, drives an expensive car and is very rich. In contrast, Dianka becomes an eternal victim, beaten, humiliated and abused. In the final scenes – robbed of “the last thing she has left: shame” ( 16 on page 315) – she ends up at a railway station among beggars and has the fantasy – in the final scene – of being a piece of rubbish that is pushed to the East by the big rubbish man along with other colourful rubbish from the West (the rubbish man is simply the person who cleans the station, but in Dianka’s fantasy he is almost a god). So it’s worth looking at the characters of *Twenty-Five* as two sides of the same traumatised subject. A subject split in two: one that serves in order to survive (and is the victor), and one that is a victim and must be distanced from in order to survive.

The rules of this split are written down very precisely in the “Decalogue of the Whore” by the autodiegetic narrator. This is the key moment of the text. At the same time: self-therapy, survival rules and almost an existential credo. Here are some of its points:

1. You are your own creation; 2. You are a god to yourself; 3. You are only interested in your own self-interest; 4. You do not sacrifice yourself either for other people or for society [...] 6. You are evil. As you can you steal, lie, all methods allowed to enrich yourself; 7. You save money for luxuries and luxury trips, because only such boys can love the rich and take them on the yacht of their lives; 8. You trust no one, no Pavo, NO ONE; [...] 10. You have no understanding for the weaknesses of others, but especially for your own. ( 16 on page 154–155)

While this is the decalogue of the main character in *Twenty-Five*, i. e. the rules for survival in the sex market, they can be understood in a metaphorical sense as general rules for success in neoliberal capitalism. Most of the points could be taken without modification from both Blake Caringtton from *Dynasty* and – even more! – Logan Roy from *Succession*.

The traumatic significance of *Twenty-Five* as an autofictional spin-off of *Lovetown* deconstructs the fundamental binaries of the earlier novel and their American-centric interpretations, marking “gays versus queers” as normalising and anti-normalising identity machines. These binaries are perhaps simply much more anchored in the Eastern European context of their time. They point to the East/West distinction as an axis of power relations that has a clearly economic (but also derivative) dimension; it can be understood in terms of Immanuel Wallerstein’s world systems analysis as the relationship between the centre and the semi-periphery of global capitalism ^17^. In other words, even if the biographical dimension of *Twenty-Five* only partially comes into play, Michał Witkowski’s words at the time of *Lovetown*’s release are perfectly understandable: “I hate the word gay, which I associate with Steven from *Dynasty*” ^11^. From the perspective of the experiences he describes in his spin-off, “gay” must have a bad connotation. He is a wealthy Western European – a German, Swiss or Austrian – who buys sex from young Eastern Europeans by exploiting his cultural and economic privileges. From the perspective of such a subject looking at a fantasy screen in the 1990s, i. e. a TV screen showing *Dynasty* and emancipatory narratives (in the guise of gay Steven), and thus from a position located within the framework (according to Polish anthropologist Michał Buchowski’s term) of “neoliberal post-socialism”, he must see one thing above all – falsity. And this has little to do with normalisation politics, but reflects a broader issue concerning the power relations between the centre and the so-called second world ^18^. And indeed – in any reflection on identity and queer politics in Poland and Eastern Europe, this distinction should be at the forefront. The ‘gej-ciota’ pair may prove to be much more synonymous and evolutionarily related than American-centred interpretive matrices suggest.

What are the further adventures of the autofictional hero of Michal Witkowski’s prose? What are the other sequels to *Lovetown*, *Twenty-Five* and “Practitioners of Non-Normative Sexuality”, which challenge the regime of normalisation? Shortly after the publication of *Twenty-Five*, Witkowski aficionados were able to buy his new autofictional novel entitled *One Hundred Dogs for Killing a Crab. A Cuban Diary*. This time, however, it was not available in an online bookshop, but could only be purchased directly from the author as a PDF file. This gesture can be understood as a challenge to the (capitalist) publishing market and at the same time as a manifesto of artistic integrity. The author wants to write what he likes, regardless of the economic conditions and the rules of the market.

Witkowski is thus at a completely different point in his literary career. The success of *Lovetown* put him on the literary map of Poland, opened the door to award nominations and led to an avalanche of translations into more than a dozen languages. The author has written other books, including autobiographies and crime novels, which have brought him commercial success and recognition. According to his maxim, which he wrote down in his autobiography entitled *Wiara* ( *Faith*), there are only two types of people: those who are on stage and those who watch them. As an eccentrically dressed fashion expert who travelled through the tabloids, he quickly found himself in show business. One can even assume that he became a performer: There was a clear continuity between his literary alter ego (Michal the man of letters) and a media personality who sometimes looked like an early Lady Gaga, which can be seen as an expression of artistic strategy. There were also scandals, and Michal Witkowski became the *enfant terrible* of the Polish literary scene. He epitomises the role of a homosexual artist who evaluates reality from his possessive (in terms of cultural and economic capital) perspective. And it is precisely this perspective that is reflected in his *Cuban Diary*.

In *Cuban Diary*, we find many elements that we recognise from his earlier works, which revolve around Michaska wanting to escape “this stupid Poland, these literary critics, reviews, nominations or non-nominations for awards, these women from publishing houses [...] and skinny guys who invite [me] to poorly paid academic conferences” ( 19 on page 47). And Michał does indeed go with his best mate Kurt to a luxury hotel in Cuba for a long holiday there, primarily for the purpose of sex tourism. And this sets the fairly regular and repetitive rhythm of the story. But why Cuba in particular? Well:

We go to the communism because the attraction for us is poverty, and for a Pole all the more so! That now I’ll be like a gringo, like a bourgeois treated, although I stuck out in lines for meat, now I’ll be meat myself [...] I’ll be the one here <from *Eneref*>! Chewing gum will be demanded of me, my Prada shoes (taken from Scotland) will be looked at with desire! ( 19 on page 23)

It is worth noting that *Eneref* is the (now obsolete) Polish colloquial abbreviation for the German Federal Republic (Niemiecka Republika Federalna, NRF) – the object of Polish secretive desires, mainly of an economic nature. The protagonist of the novel therefore immediately places himself in a dominant, almost colonising position.

However, if one were to look at the *Cuban Diary* from a slightly more psychoanalytical perspective, everything falls into place in an understandable way. Michał chooses Cuba (and not Thailand) for a very specific reason – because here he finds one of the last places on the globe that is called “communism”. And this is exactly where he wants to buy sex. So he returns to the place of trauma, but from the other side, so to speak. He returns from a position of strength, he’s in charge. He is no longer Dianka from Eastern Europe selling sex on Karlplatz. This time he is the one buying sex from men who represent “communism” – perhaps even a little too obsessively. As if it was not about sex, but about power and a sense of omnipotent self-determination and empowerment. Yes, he’s still not keen on “gays” (here in the form of a hyper-sensitive French couple), although he’s actually not much different from them. For in his case, too, the opposition “gay versus queer” (gej/ciota) was from the outset much more immersed in an Eastern European context than could be inferred from translating it into globally recognizable American distinctions. Indeed, Michał is probably closest to the rather conservative figure of the homosexual aristocrat. But his queer art, including that of the *Cuban Diary*, remains dark, expressing the desires, complexes and fantasies of the inhabitants of the semi-periphery of global capitalism.

## Conclusion

*Lovetown* ultimately proves to be a political novel – despite Michał Witkowski’s own declarations that he is not interested in politics. Although Witkowski repeatedly claims this, he has nevertheless introduced a radically new vision of male homosexuality into Polish public discourse, one that can be described as ‘gay camp’, non-binary or even transgender (the novel can be interpreted through all these lenses). In this sense, *Lovetown* functions as a medium of public — and thus political — intervention. The novel’s formal features, such as its use of colloquial language and slang, as well as its fragmented and heterogeneous plot structure, further reinforce its political resonance.

At the same time, the ciota/gej dichotomy at the centre of the novel hints at an additional layer of political meaning, especially when considered in the context of Witkowski’s later works *Twenty-Five* and *One Hundred Dogs for a Crab: A Cuban Diary*. When translated into the opposition queer versus gay (or LGBT) familiar from American theory, the ciota/gej distinction can easily be misinterpreted. This is because the translation distorts the meaning of the term, which is deeply rooted in the Eastern European context and does not have the clear identity-based connotations that are common in the West. In Witkowski’s work, as a deconstructive analysis shows, this distinction has an economic undertone that refers to the East-West divide. *Lovetown* can therefore be read as a political novel: as a critique of post-socialist neoliberalism.

## Ethics and consent

Ethical approval and consent were not required.

## Data Availability

No data associated with this article.
