# Supplementary material for: From Poland with love: Politics of queerness in the time of neoliberal post-socialism (The case of Michał Witkowski)
Source: Open Res Eur. 2025 Jul 8;5:179. [Version 1] doi: 10.12688/openreseurope.19923.1 (PMC13555006; doi:10.12688/openreseurope.19923.1)
Supplement: Peer review history

# Reviewer report: Reviewer response for version 1

**DOI:** 10.21956/openreseurope.21551.r77426

**Published:** 2026-07-21

**Authors:** Tomasz Basiuk

**Affiliations:** 1 University of Warsaw, Warsaw, Poland

Blazej Warkocki’s reading of Michal Witkowski 2005 *Lovetown *situates it in the context of two subsequent autofictions developing themes from the earlier novel; it also polemically engages the standard reading of *Lovetown, *exemplified here by the scholarly work of Magda Szcześniak. The critical consensus about *Lovetown *has been that it pits the pre-gay-lib homosexual ( *ciota*, translated as ‘queer’) against the post-gay-lib, 'normalized' homosexual ( *gej*, the Polish spelling of ‘gay’), while nostalgically gazing back at the queer past. According to Warkocki, this reading misses Witkowski's point in two distinct ways. One: ‘faggot’ would be a more accurate translation of *ciota* than ‘queer’ because Witkowski’s aging homosexuals are gender-bending: they address each other as women and engage in various campy practices. (The Polish word ‘ciota’ has similar connotations to the German *tunte*.) Reading the novel in terms of the gay-lib caesura seems outdated; it would be more appropriate to apply non-binary or trans labels to the novel’s main characters. Such re-visioning of the terms would be more aligned with state-of-the-art approaches in queer studies some two decades after the novel’s release. This interesting argument is only partly developed, however.

 The main thrust of Warkocki’s polemical reading lies elsewhere. He notes that the gay-lib caesura is imported from the West (and perhaps specifically from the U.S.), and that it unfortunately pays no mind to the context of Poland’s post-1989 transition from state socialism to a democracy and a market economy. Warkocki describes this new economy as neoliberal, noting that, from the 1990s onwards, Poland is no longer a part of the now-disappeared ‘Second World’ but lies somewhere in the semi-periphery of global capitalism (as per Immanuel Wallerstein’s formula). Highlighting this broader historical context of *Lovetown*, two sequels to the novel – the officially released *Fynf und Cwancyś *(Twenty-Five), which is a story of East European youths prostituting themselves in Vienna, and the self-published *Sto psów za zabicie kraba. Dziennik kubański *(One Hundred Dogs for Killing a Crab. A Cuban Diary), which is styled as a travelogue of a sex tourist visiting Cuba – render all the more palpable the painful divide between the opulence afforded by a market economy and the relative destitution resulting from an inefficient, state-run economy.

 Warkocki notes that grasping the East/West divide solely in terms of gay liberation, and thus in terms of the relative belatedness of Central and Eastern Europe with respect to LGBTQ rights – the perspective which informs Szcześniak’s reading of *Lovetown *– obscures the economic gap between the aging protagonists who fondly recall the state-socialist era (a time of their youth) and the younger characters adapting to the emerging market economy with much greater ease and profiting from the country’s post-1989 political and economic transition. He argues that this oft-overlooked aspect of the novel makes it political in a way to which an exclusive focus on gay liberation cannot do justice, even if, historically, gay rights discourse emerged in Poland approximately at the time of this broader economic and political transition.

 Warkocki’s argument is persuasive, even if Witkowski does not himself appear to take a stance vis-à-vis the post-1989 transition to market economics, but merely makes this transition a key element of his writing. To my knowledge, at least one reviewer (Kinga Dunin) has raised a similar point at the time that *Lovetown *was first published. Nonetheless, Warkocki’s intervention is worthwhile given the prevailing manner of reading *Lovetown *as solely organized around the gay/queer axis.

 There are a few loose ends to the article as it now stands. One has been mentioned above: it would be interesting to see the argument about ‘faggots’ as non-binary or transgender (rather than simply homosexual) developed further. Another loose end is the criminality found in *Lovetown *and euphemistically glossed over by Szcześniak: the novel’s protagonists engage in sex with a drunken straight man who is unable to express consent due to his intoxication, and they also appear to kill a man. While this disturbingly transgressive aspect of *Lovetown* may be tied to *Wir Kinder…*, which Warkocki brings up as a crucial, if dark, intertext for Witkowski’s novel, he leaves this argumentative strand underdeveloped.

 Finally, the article in its present form could use some language editing to make it easier to read.

If any, are all the source data and materials underlying the results available?

No source data required

Is the work original in terms of material and argument?

Yes

Is the work clearly and cogently presented?

Yes

Is the argument persuasive and supported by evidence?

Yes

Does the research article contribute to the cultural, historical, social understanding of the field?

Yes

Does it sufficiently engage with relevant methodologies and secondary literature on the topic?

Partly

Reviewer Expertise:

contemporary American literature, life writing, queer studies

I confirm that I have read this submission and believe that I have an appropriate level of expertise to confirm that it is of an acceptable scientific standard.

# Reviewer report: Reviewer response for version 1

**DOI:** 10.21956/openreseurope.21551.r59894

**Published:** 2025-09-16

**Authors:** Purwarno Purwarno

**Affiliations:** 1 Universitas Islam Sumatera Utara, Medan, Indonesia

1. Summary of the Article:

 This article examines Michał Witkowski’s *Lubiewo* ( *Lovetown*, 2005) and subsequent works ( *Twenty-Five*, 2015; *One Hundred Dogs for Killing a Crab*, ca. 2018) as central texts in post-socialist queer literature. Warkocki’s main claim is that *Lovetown* is an “unconsciously political novel” despite the author’s repeated disavowal of political intent. By foregrounding the local distinction between *gej/ciota* (gay/faggot), Warkocki critiques Western readings that too easily translate this into the American theoretical binary of “gay versus queer.” He argues that such translations erase the specific Eastern European socio-economic and cultural context—particularly Poland’s neoliberal transformation and the persistence of hegemonic masculinity.

 Through close readings and contextual analysis, Warkocki situates Witkowski’s autofiction within larger dynamics of East–West relations, semi-peripherality, and global capitalism. He engages with theorists such as Jacques Rancière, Maria Janion, and Immanuel Wallerstein to frame the novels as both cultural texts and critiques of post-socialist modernity. The essay ultimately positions Witkowski’s work as a key contribution to queer cultural history in Poland and Eastern Europe.

 2. Evaluation of Key Criteria

 Originality of material and argument – Yes

 The article is original in both material and argument. It goes beyond existing scholarship by:

- Analyzing not only *Lovetown* but also Witkowski’s lesser-discussed works ( *Twenty-Five* and the privately circulated *Cuban Diary*).
- Offering a fresh theoretical framing of *Lovetown* as a political text despite the author’s own disavowals.
- Critiquing the “Americanization” of queer theoretical frameworks, instead situating the discussion within post-socialist neoliberal transformation.

This layered approach makes the work a novel and significant intervention in both queer literary studies and Eastern European cultural history.

 Engagement with methodology and secondary literature – Partly

 The article demonstrates strong engagement in some respects:

- It makes effective use of deconstructive analysis, Rancière’s aesthetics and politics, and Wallerstein’s world-systems framework.
- It dialogues with existing Polish scholarship, especially Magda Szczęśniak’s *Normy widzialności*, by acknowledging its insights while also critiquing its blind spots.
- It references important figures in queer and cultural theory (Judith Butler, Maria Janion, Agata Pyzik).

However, gaps remain:

- Transgender studies and vocabulary are largely absent. Although the article itself notes this, it does not fully integrate cis/trans or non-binary frameworks that could deepen the analysis, especially given Witkowski’s characters’ fluidity.
- Comparative queer methodologies beyond Poland are underutilized. The critique of American queer frameworks is valuable, but the article might benefit from engaging with parallel debates in other post-socialist or semi-peripheral contexts (e.g., Balkan, Baltic, or Latin American queer studies).

Constructive suggestion:

 To strengthen the methodological foundation, the author should incorporate transgender studies concepts where relevant and broaden the comparative scope beyond Poland and the U.S. This would prevent the analysis from appearing selective and make the argument more robustly grounded in contemporary queer scholarship.

 Clarity and cogency of presentation – Yes

 The article is well-structured, progressing logically from introduction to analysis to conclusion. Key terms are clearly defined, and secondary literature is woven in seamlessly. The writing is academic yet accessible, making the complex interplay of theory and textual analysis intelligible to readers across disciplines.

 Availability of source data and materials – No source data required

 As a work of literary and cultural analysis, the article does not require datasets. All primary sources (Witkowski’s novels and essays) and secondary materials are properly cited.

 Contribution to cultural, historical, and social understanding – Yes

 The article contributes significantly to the field by:

- Expanding cultural understanding of queer Polish literature.
- Providing historical context by linking Witkowski’s novels to the neoliberal transformation of post-socialist Poland.
- Offering social insight into the dynamics of masculinity, queerness, and semi-peripherality in Eastern Europe.

It enriches both queer studies and post-socialist cultural history, making it relevant to scholars in multiple disciplines.

 3. Points That Must Be Addressed for Scientific Soundness

 To ensure the article is fully rigorous and scientifically sound, I recommend the following:

1. Engage with transgender studies frameworks – At minimum, the article should clarify why cis/trans or non-binary categories were not systematically employed, beyond acknowledging their absence. Ideally, integrate these perspectives into the analysis of Witkowski’s characters, who are rarely wholly cisgender.
2. Expand comparative scope – The critique of American-centric queer theory would be strengthened by reference to other post-socialist or semi-peripheral queer contexts (e.g., work from the Balkans, Baltics, or Latin America). This would demonstrate that the article is not replacing one narrow framework with another but situating Polish queer literature in a broader comparative field.
3. Clarify terminology – Certain terms (e.g., “ciota”) are potentially offensive. While their historical and cultural specificity is crucial, the author should provide additional reflection on the ethics of retaining such terminology, and perhaps propose parallel contemporary vocabulary for clarity.

4. Overall Recommendation

 The article is well-argued, original, and contributes meaningfully to cultural and queer studies. It is clearly presented and does not require empirical data. However, for it to be methodologically comprehensive and scientifically sound, the author should:

- More explicitly incorporate transgender studies,
- Extend the comparative framework beyond Poland/U.S., and
- Reflect critically on terminology.

With these revisions, the article will be both intellectually rigorous and socially responsible, further cementing its contribution to the study of queer literature in post-socialist contexts.

If any, are all the source data and materials underlying the results available?

No source data required

Is the work original in terms of material and argument?

Yes

Is the work clearly and cogently presented?

Yes

Is the argument persuasive and supported by evidence?

Yes

Does the research article contribute to the cultural, historical, social understanding of the field?

Yes

Does it sufficiently engage with relevant methodologies and secondary literature on the topic?

Partly

Reviewer Expertise:

Literature, Cultural Studies, English Studies

I confirm that I have read this submission and believe that I have an appropriate level of expertise to confirm that it is of an acceptable scientific standard.
